# The Prolongation of Nitrous Oxid Anesthesia

**Published:** 1898-12

**Authors:** Herbert J. Paterson


ARTICLE IV.

### THE PROLONGATION OF NITROUS OXID ANESTHESIA.

HERBERT J. PATERSON, M. A., M. F. CANT., F. R. C. S., LOND.

At this domonstration the anesthetic was given for the removal of a living pulp previous to crowning. The anesthesia was perfectly satisfactory and was officially returned as four minutes fifty-five seconds, but could have been considerably prolonged had not the operation been finished.

In describing the method, Dr. Paterson said : "Before domonstrating to you an apparatus for prolonging nitrous oxid anesthesia on the principle of that lately introduced by one of your oldest members, Mr. Alfred Coleman, I will with your permission very briefly call your attention to one point in connection with this important subject. Even now many regard it dangerous to prolong nitrous oxid anesthesia, but I am convinced that such fears are groundless, and I claim to have demonstrated that nitrous oxid anesthesia may be maintained as long as anesthesia by any other agent, with greater safety and more comfort to the patient than anesthesia by any other means. In the *British Medical Journal*, of January 22, 1898, I recorded a series of cases in support of this contention, the longest case in that series lasting sixty-five minutes. Since that time I have had several cases lasting considerably more than one hour, and in one of the cases anesthesia was maintained for two hours and ten minutes. The patient suffered from absolutely no collapse, and regained consciousness within four minutes and was able to have his tea within half an hour. I believe these are by far the longest cases on record, the longest recorded cases I have been able to find lasting eighteen minutes. In none of these cases were any disquieting symptoms observed, and in many of them the state of the heart during the administration was carefully investigated by one of my medical colleagues, no evidence of dilitation or irregularity being detected. The cases were watched for the next three days for the supervention of after-effects, none being observed. Many attempts have been made to prolong nitrous oxid anesthesia during operations about the mouth, and I, in common with others, have tried various devices, among them that of passing the gas into the naso-pharynx by means of a catheter introduced through one nostril. This method has such insuperable objections that I have finally given it up. The apparatus I show to-day is a modification of that shown by Mr. Coleman, and consists of a nose-piece adapted to fit accurately over the nose and connected by a large tube, passing over the patient's head, with an ordinary gas bag, a two-way stopcock intervening. The nose-piece is made of copper, and as the air pad is detachable, this part can be readily sterilized by boiling. In using the apparatus the nose-piece is placed *in situ*, an air padded face-piece of large size, having an expiratory valve, is placed over the face, and the gas turned on. When anesthesia is established the face-piece is removed and the operation proceeded with. The patient is now breathing gas by the nose and a certain amount of air by the mouth, and the proportions of these can be regulated by increasing or decreasing the pressure of gas in the bag. I think I shall be able to further simplify the apparatus, and the necessity of this large and cumbersome tube can I think be done away with by having two smaller tubes, one on either side of the patient's head. By a simple arrangement the apparatus can be used as an ordinary gas machine or for giving gas and air.

In giving gas for prolonged periods I have for some time been in the habit of warming the gas after it leaves the bottles, to counteract the very considerable fall of temperature which ensues when a continuous stream of gas is allowed to escape from the bottle. If this be not done, not only may the cold gas irritate the air passages, but I am strongly convinced, for reasons in which I will not enter now, that it is this coldness of the gas which is answerable for the retching and vomiting which sometimes occur even in patients who have been properly prepared, I may say that so far the results I have obtained by this method have been highly satisfactory. In addition to the advantage gained by the practically unlimited time at the disposal of the operator, and the elimination of the use of ether in dental operations, and I maintain that this is the best method of administering gas for ordinary dental extractions. The ordinary method of dosing the patient with gas to the utmost limit, so as to maintain the anesthesia as long as possible after the removal of the face-piece, is most unscientific and wrong in principle. By this method the anesthesia need not be pushed so far, the anesthesia is carried only to the stage of unconsciousness, and the patient maintained gently under the influence of the gas without pushing it to an extreme degree. If I may be allowed to make an analogy, the ordinary method compared with this method is as if an express train were to start on its journey with an extra tender loaded with water, instead of, as is the modern custom, taking up the water it requires while going on its way at full speed. So by this method the gas is given as required, instead of giving an overdose at the commencement.—*Journal British Dental Association.*

